# Carbon Dots Extracted from the Plant *Gardenia jasminoides* Ameliorates Ischemia–Reperfusion Injury

**DOI:** 10.3390/ph18060870

**Published:** 2025-06-11

**Authors:** Liyang Dong, Haojia Zhang, Kai Wang, Chunyu Wang, Yiping Wu, Wei Shao, Kunjing Liu, Xin Lan, Jinhua Han, Jialin Cheng, Changxiang Li, Xueqian Wang, Fafeng Cheng, Qingguo Wang

**Affiliations:** Chinese Medicine College, Beijing University of Chinese Medicine, Beijing 102488, China; dly1613@163.com (L.D.); zhanghj61@163.com (H.Z.); hello_wkwangkai@163.com (K.W.); 20230931020@bucm.edu.cn (C.W.); wuyp199711@163.com (Y.W.); bjzyydxsw@163.com (W.S.); 20230941024@bucm.edu.cn (K.L.); laci_97@163.com (X.L.); 20230931035@bucm.edu.cn (J.H.); linjiachengjcl@163.com (J.C.); changxiang1202@163.com (C.L.); shirlyding@163.com (X.W.)

**Keywords:** ischemic stroke, *Gardenia jasminoides*, carbon dots, inflammation, oxidative stress

## Abstract

**Background:** Ischemic stroke (IS) is probably the most important acute serious illness, where interdisciplinary approach is essential to offer the best chance for survival and functional recovery of patients. Carbon dots (CDs) with multifaceted advantages have provided hope for development brand-new nanodrug for treating thorny diseases. **Methods:** This study developed a green and environmentally responsible calcination method to prepare novel *Gardenia jasminoides* Carbonisata (GJC-CDs) as promising drug for ischemic stroke treatment. **Results:** In this work, we isolated and characterized for the first time a novel carbon dots (GJC-CDs) from the natural plant *G. jasminoides*. Results displayed that green GJC-based CDs with tiny sizes and abundant functional groups exhibited solubility, which may be beneficial for its settled biological activity. The neuroprotective effect of carbon dots from *G. jasminoides* were evaluated using the classical middle cerebral artery occlusion (MCAO) model. Assessing the infarct volume content of the ischemic cerebral hemisphere and determining the serum tumor necrosis factor-α (TNF-α), interleukin-1β (IL-1β), interleukin-6 (IL-6), interleukin-10 (IL-10), reduced glutathione (GSH), superoxide dismutase (SOD), and malondialdehyde (MDA) levels of the mice in each group, it was evident that pre-administration of the drug by GJC-CDs significantly reduced the infarct volume as well as attenuated inflammatory responses and excessive oxidative stress in MCAO mice. Furthermore, in vitro cellular experiments demonstrated that GJC-CDs have good biosafety and anti-inflammatory and antioxidant capacity. **Conclusions:** Overall, GJC-CDs performs neuroprotective effect on cerebral ischemia and reperfusion injury, which not only provides evidence for further broadening the biological application of acute ischemic stroke but also offers novel strategy for the application of nanomedicine to treat acute diseases.

## 1. Introduction

Stroke is defined as a sudden interruption of focal or global neurological symptoms due to a blockage of a certain brain artery or rupture of a cerebral vessel [1]. However, ischemic stroke is the main cause of acquired physical disability and the most common cause of death worldwide [2]. As the population ages and the prevalence of risk factors like hypertension, hyperlipidemia, and diabetes continues to rise and remains poorly controlled, the burden of stroke in the world is also increasing [3]. Hence, pathogenesis of stroke and recovery strategies are urgently needed in clinical practice.

Localized ischemic infarction of the brain results in the release of inflammatory factors (damage-associated molecular patterns, DAMP) that, on one side, locally activate immunocompetent cells. On the other side, DAMP release to the blood circulation can promote the recruitment of circulating immune cells to the area of infarction and activate a complex peripheral immune response to stroke [4]. Inflammatory immune cells exacerbate ischemic neuronal injury after the onset of brain ischemia; however, some of the immune cells thereafter change their function to neuroprotection. The recovery processes after cerebral infarction require close interactions between the immune and nervous systems through various mechanisms. Thus, the brain restrains its own inflammation after injury via the immune system, which provides an innovative therapeutic strategies for stroke patients [5].

Oxidative stress refers to an imbalance between the production of reactive oxygen species (ROS) and the body’s antioxidant defense mechanisms [6]. Following acute cerebral infarction, intense neuroinflammation occurs within the brain, which in turn activates inflammatory pathways that promote the excessive generation of free radicals, particularly ROS [7]. During this inflammatory process, immune cells such as microglia become activated and release various free radicals, including superoxide anions (O_2_^−^) and hydrogen peroxide (H_2_O_2_) [8,9]. The accumulation of these reactive molecules within cells leads to structural and functional damage to cellular components such as membranes, proteins, and DNA. This cascade ultimately results in oxidative stress, which further amplifies neuronal injury and tissue damage.

Although the pathogenesis of ischemic stroke is not yet fully clear, studies have shown that excessive oxidative stress plays an important role in the development of this disease [10]. Excessive oxidative stress participates in cerebral ischemia injury through many ways, which damages the normal function of brain tissue, causes irreversible damage to neuronal cells, ultimately leads to cell apoptosis and necrosis [11]. Oxidative stress accompanies several pathological processes and results from increased ROS production, mostly via oxidative phosphorylation in the mitochondria. When cerebral ischemia occurs, excessive oxidative damage contributes to cytochrome c release and mitochondrial permeability transition pore (MPTP) opening, which further promotes mitochondrial damage-associated molecular patterns, including the activation of the systemic inflammatory responses [4].

The main strategies of ischemic stroke treatment include salvaging the volume of hypoperfused and ischemic penumbra, where the tissue surrounding the infarcted core is still viable. Indeed, remarkable progress has been made in the recovery of patients with acute ischemic stroke in the past several years, with the widespread application of stroke treatment methods and evidence of the efficacy of intravenous thrombolysis via recombinant tissue plasminogen activator (rtPA), establishing the value of endovascular thrombectomy [12]. However, numerous animal trials have failed to show efficacy of drugs that modulate one or more of these mechanisms in animals with ischemic stroke, despite promising experimental data [13].

Recently, nanotechnology has been an innovative topic of scientific research and has had a huge boost to the development of medicine, including nano-diagnostics, nano-biosensors and nano-biomaterials [14,15]. Nanotechnology has made significant advances in the diagnosis and therapy of stroke in recent years. In one study, Zhao reported the use of nano-liposomes to deliver basic fibroblast growth factor (BFGF) to the brain, effectively reduced infarct volume [16]. An impressive and emerging field is the use of magnetic iron oxide nanoparticles (MIONPs) for the delivery of drugs into the brain and for monitoring delivery efficacy via magnetic resonance imaging (MRI) or magnetic particle imaging (MPI) [17,18].

Carbon dots (CDs), a novel carbon-based bioactive nanomaterial with a characteristic size of less than 10 nm and containing abundant organic functional groups, have been reported in 2004 and have aroused growing attention in various applications including physics, chemistry, and medicine, due to their excellent properties such as high bioactivity, photoluminescence property, high biocompatibility, and low toxicity [19]. Consequently, based on green synthesis process, inexpensiveness, ability to reduce ROS, and immunoregulation and autophagy regulation ability, natural herbaceous plant-derived CDs naturally attracted further attention in modern research, which can be an efficient strategy to improve the complexity, biotoxicity, and low utilization of natural medicines [20]. Recent studies has confirmed a number of CDs have the promising strategies to serve as nanomedicine in stroke [21]. In one study, a new species of carbon dots derived from Crinis Carbonisatus (CrCi-CDs) was separated and identified, the neuroprotective effect of carbon dots was evaluated using the middle cerebral artery occlusion model [22]. Thus, these results provide sufficient evidence for natural drug-derived CDs for the treatment of ischemic stroke.

*G. jasminoides* (*G. jasminoides* J.Ellis), named zhizi in Chinese, is a traditional herb that has been used for thousands of years and is documented in the classical Chinese medicine antiquarian book “Taiping Holy Prescriptions for Universal Relief” on the use of charcoal, the overly carbonized powder of *G. jasminoides* had shown the amelioration in the cerebral ischemia, it were still employed in the clinical therapy. Herein, a novel CD (GJC-CD) prepared by the green and one-step calcination method using *G. jasminoides* as a carbon source and its ameliorative effect on middle cerebral artery occlusion-induced acute cerebral ischemic infarction have been studied for the first time. In addition, the physical morphology and optical properties of GJC-CDs as well as the functional groups and ligand bonds on their surfaces were analyzed and then evaluated for safety by cellular experiments.

## 2. Results

### 2.1. Analysis of GJC-CDs

**Figure 1:**
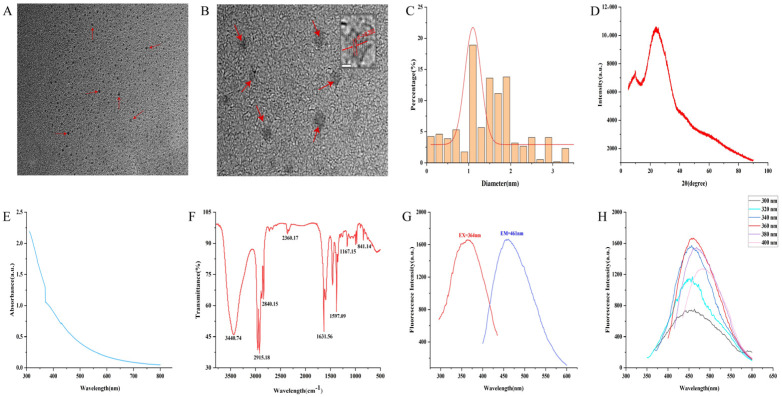
Morphological and optical characterizations of GJC-CDs. (**A**) Transmission electron microscopy (TEM) images (30 k×, 20nm, arrows represent GJC-CDs observed under electron microscopy). (**B**) High-resolution transmission electron microscopy (HRTEM) images (500 k×, 2nm, arrows represent GJC-CDs observed under electron microscopy). (**C**) Particle size distribution histogram. (**D**) X-ray diffraction pattern. (**E**) Ultraviolet–visible spectrum. (**F**) Fourier transform infrared spectrum. (**G**) Fluorescence spectra for excitation and emission. (**H**) Fluorescence spectra of GJC-CDs with different excitation wavelengths.

The morphology and particle size distribution of GJC-CDs were characterized using transmission electron microscopy (TEM) and high-resolution transmission electron microscopy (HRTEM). TEM analysis (Figure 1A) revealed that the GJC-CDs adopted a uniform quasi-spherical morphology with a monodisperse distribution, exhibiting particle sizes ranging from 0.2 to 4.5 nm (Figure 1C). HRTEM imaging (Figure 1B) further demonstrated a distinct lattice spacing of 0.201 nm in the GJC-CDs, corresponding to the (100) lattice planes of graphite, thereby indicating structural similarities to the crystalline framework of graphite. The amorphous nature of the GJC-CDs was corroborated by XRD analysis, where a broad diffraction peak observed at 2θ = 24.05° (Figure 1D) confirmed their predominantly amorphous carbon phase. This finding aligns with the structural features observed in the HRTEM images.

The luminescent characteristics of GJC-CDs were systematically evaluated. UV-Vis absorption spectroscopy (Figure 1E) revealed a featureless broad absorption profile with a weak peak centered at 370.22 nm, attributed to n-π* electronic transitions in conjugated C=O/C=N bonds and aromatic sp^2^ domains. Fourier-transform infrared (FTIR) spectroscopy (Figure 1F) was employed to probe the surface functional groups. The spectrum exhibited a broad band at 3440.74 cm^−1^ corresponding to O–H/N–H stretching vibrations. Peaks at 2915.18 cm^−1^ and 2840.15 cm^−1^ were assigned to C–H stretching modes in –CH₃ and –CH₂ groups. A prominent absorption at 1631.56 cm^−1^ was characteristic of C=O stretching. A peak at 1597.09 cm^−1^ was associated with C–N vibrations. A distinct signal at 1167.15 cm^−1^ was indicative of aromatic C–O bonds. These observations collectively confirm the presence of multifunctional surface groups, including carbonyl, hydroxyl, and amino moieties. Fluorescence studies demonstrated excitation-dependent emission behavior. Under 364 nm excitation, GJC-CDs exhibited a maximum emission peak at 461 nm (Figure 1G). Notably, when the excitation wavelength was incrementally increased from 300 nm to 400 nm (20 nm intervals), the emission maximum underwent a progressive red shift accompanied by an initial intensity enhancement followed by attenuation (Figure 1H), a hallmark of excitation wavelength-responsive luminescence.

**Figure 2:**
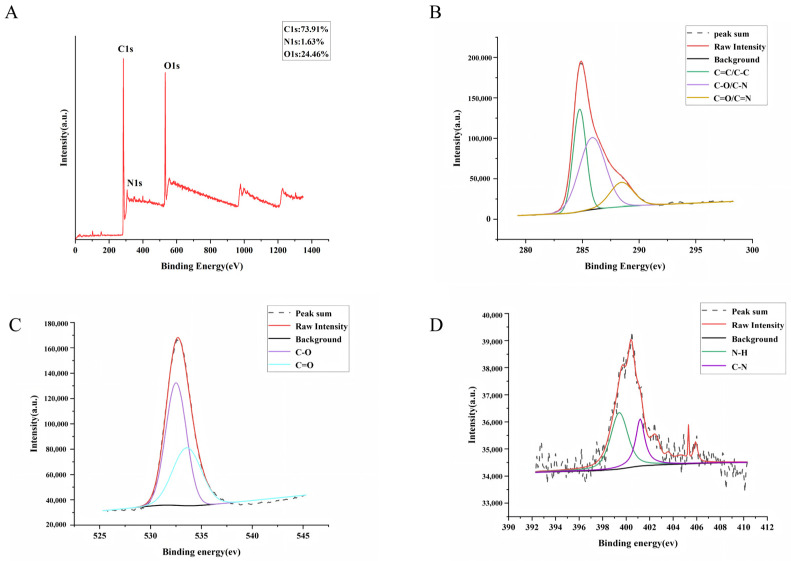
The surface composition and elemental analysis of the prepared GJC-CDs by XPS. (**A**) X-ray photoelectron spectroscopy survey of GJC-CDs. (**B**) C1s. (**C**) O1s. (**D**) N1s.

The surface elemental composition and chemical bonding of GJC-CDs were determined by x-ray photoelectron spectroscopy (XPS), as shown in Figure 2A. The results of the study indicate that the substance is mainly composed of three elements, C (73.91%), O (24.46%) and N (1.63%). The C1s (Figure 2B) spectra show distinct peaks at 284.74 eV, 285.87 eV, and 288.44 eV, indicating the presence of coordination bonds such as C=C/C–C, C–O/C–N, and C=O/C=N. The O1s (Figure 2C) spectra show distinct peaks at 532.5 eV and 533.58 eV, indicating the presence of coordination bonds such as C–O and C=O. The N1s (Figure 2D) spectra show distinct peaks at 399.76 eV and 400.67 eV, indicating the presence of coordination bonds such as N–H and C–N.

### 2.2. GJC-CDs Reduce Cerebral Infarct Volume and Improves Neurological Function in MCAO Mice

**Figure 3:**
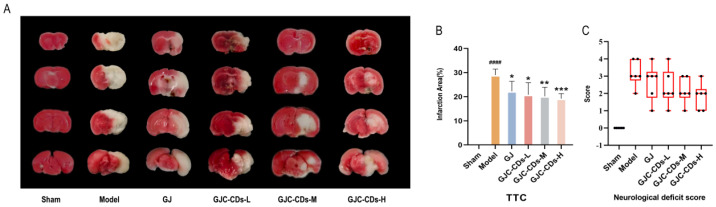
Effect of GJC-CDs on cerebral infarct volume and neurologic function in MCAO mice. (**A**) TTC staining. (**B**) Percentage of infarct volume (*n* = 6). (**C**) Zea-Longa scores (*n* = 6, each dot represents the neurological functioning score of an MCAO mouse). Data were represented as means ± SD. ^####^ *p* < 0.001 compared with the Sham group, * *p* < 0.05, ** *p* < 0.01 and *** *p* < 0.001 compared with the Model group.

The succinate dehydrogenase enzyme in the mitochondria of normal brain tissue reacts with the TTC dye to produce a red coloration. In contrast, infarcted brain areas appear pale due to the inability to undergo this reaction. As illustrated in Figure 3A, the remaining groups exhibited disparate infarct foci (white areas) in comparison to the sham-operated group. As demonstrated in Figure 3B, in comparison to the sham-operated group, the cerebral infarct volume was significantly increased in the model group (*p* < 0.0001), whereas both the *G. jasminoides* group and all of the GJC-CDs group demonstrated a reduction in volume to varying degrees, with the most significant improvement being seen in the high-dose group of GJC-CDs (*p* < 0.001). Furthermore, the results of the Zea-Longa scores (Figure 3C) indicated that the mice in the model group exhibited neurological and behavioral deficits. However, the neurological deficits of the experimental animals could be significantly improved by the pre-administration of GJC-CDs, showing a certain dose-dependence.

### 2.3. GJC-CDs Enhance Cerebral Blood Flow in MCAO Mice

**Figure 4:**
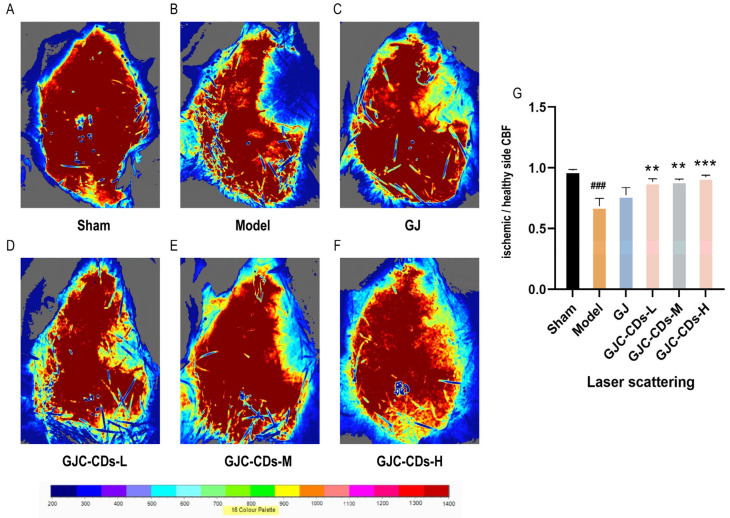
Effect of GJC-CDs on cerebral blood circulation in MCAO mice. (**A**) Sham group; (**B**) Model group; (**C**) GJ group; (**D**) Low-dose GJC-CDs group; (**E**) Medium-dose GJC-CDs group; (**F**) High-dose GJC-CDs group; (**G**) The ratio of CBF in each group of mice (*n* = 6). Data were represented as means ± SD. ^###^ *p* < 0.001 compared with the Sham group, ** *p* < 0.01 and *** *p* < 0.001 compared with the Model group.

In this study, laser scatter haemodynamic imaging was employed to quantify bilateral cerebral cortical blood flow in all mouse groups, with the objective of evaluating the impact of GJC-CDs on cerebral blood flow. As illustrated in Figure 4A, the blood flow on both sides of the brain in the sham-operated group of mice remained unimpaired, with no significant difference, indicating that bilateral cerebral blood flow was stable in the absence of cerebral ischemia–reperfusion injury. In contrast, mice in the model group (Figure 4B) exhibited a notable reduction in cerebral blood flow on the infarcted side relative to the healthy side following cerebral ischemia–reperfusion, suggesting that cerebral ischemia–reperfusion is associated with a decline in cerebral blood flow, which is a pivotal aspect of the pathological cascade associated with cerebral ischemia. It is noteworthy that following the administration of GJC-CDs (1.5, 3 and 6 mg/kg, respectively) prior to the experiment, all groups of mice exhibited a notable enhancement in cerebral blood flow (*p* < 0.01), but the effect was not significant in the GJ group (*p* > 0.05).

### 2.4. GJC-CDs Improve Intracerebral Pathological Damage in MCAO Mice

**Figure 5:**
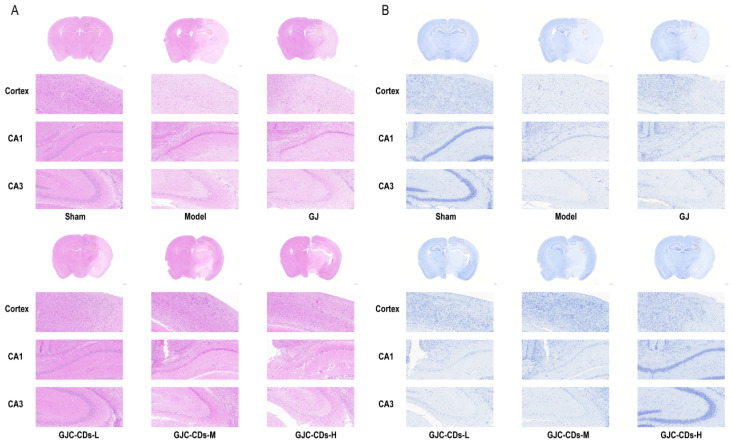
Effect of GJC-CDs on intracerebral pathological damage in MCAO mice. (**A**) Hematoxylin-eosin staining (*n* = 4). (**B**) Nissl staining (*n* = 4).

To further investigate the protective effects of GJC-CDs on the brain, we employed hematoxylin-eosin staining and Nissl staining for histopathological examination of the brain tissues of each group of mice (Figure 5). The neurons in the cerebral cortex of mice in the sham-operated group exhibited a high degree of organization, with a neat arrangement and complete structural morphology. The nuclei were round, the cytoplasm was abundant, the staining was uniform. The model group exhibited a notable reduction in the number of neurons within the brain tissue of mice. The arrangement of nerve cells was observed to be sparse and disordered, with the nuclei of the cells appearing shrunken and deeply stained. Additionally, some cells demonstrated evidence of edema and pathological ischemic damage, such as a significant increase in the perivascular gap. In contrast, the number of neurons in the cerebral cortex and hippocampal region of mice in all groups after pre-treatment with GJ and GJC-CDs increased to varying degrees. Neurons were arranged in a more orderly manner, the number of necrotic cells was significantly reduced, and the boundaries of the cells were more clearly defined. Among these outcomes, the improvement was most significant in the high-dose group of GJC-CDs (6 mg/kg). The results of Nissl staining demonstrated that neuronal Nissl’s vesicles in the cortex and hippocampal region of the mice in the sham-operated group were larger in size and number. Additionally, the cells exhibited a normal morphology and structure, with clearly defined boundaries. The number of Nissl’s vesicles in the brains of mice in the model group was significantly reduced, the cell gap was widened, the cellular morphology was swollen and irregular. However, these pathological impairments could be significantly ameliorated by pre-treatment with GJ and GJC-CDs (1.5, 3 and 6 mg/kg, respectively), with the high dose of GJC-CDs being the most efficacious.

### 2.5. GJC-CDs Inhibit Inflammation Levels in MCAO Mice

**Figure 6:**
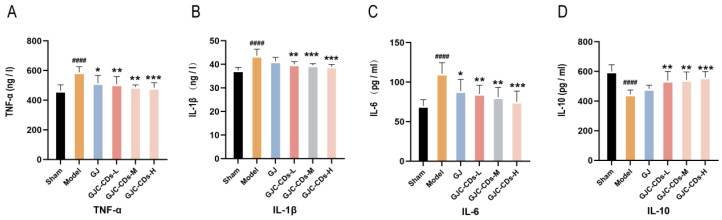
Effect of GJC-CDs on inflammation levels in MCAO mice. (**A**) TNF-α levels in serum (*n* = 6). (**B**) IL-1β levels in serum (*n* = 6). (**C**) IL-6 levels in serum (*n* = 6). (**D**) IL-10 levels in serum (*n* = 6). Data were represented as means ± SD. ^####^ *p* < 0.0001 compared with the Sham group, * *p* < 0.05, ** *p* < 0.01, and *** *p* < 0.001 compared with the Model group.

Global brain inflammation may continue to influence the evolving pathology after stroke. To investigate whether GJC-CDs are involved in the inflammatory response in ischemic stroke, we examined the levels of TNF-α, IL-1β, IL-6, and IL-10 in the serum of each group of mice. As illustrated in Figure 6A–D, the levels of TNF-α, IL-1β, and IL-6 were markedly elevated (*p* < 0.0001), and those of IL-10 were markedly reduced (*p* < 0.0001) in the model group in comparison to the sham surgery group. In contrast, pretreatment with GJC-CDs (1.5, 3 and 6 mg/kg, respectively) resulted in a significant reduction in TNF-α, IL-1β, and IL-6 levels and an elevation in IL-10 levels across all groups of mice, with the high dose of GJC-CDs demonstrating the most pronounced effect (*p* < 0.0001). In addition, levels of the anti-inflammatory factor IL-10 were increased and levels of the pro-inflammatory factor IL-1β were decreased in the GJ group compared with the model group, but none of them were statistically different from each other. (*p* > 0.05)

### 2.6. GJC-CDs Improve Antioxidant Levels in MCAO Mice

**Figure 7:**
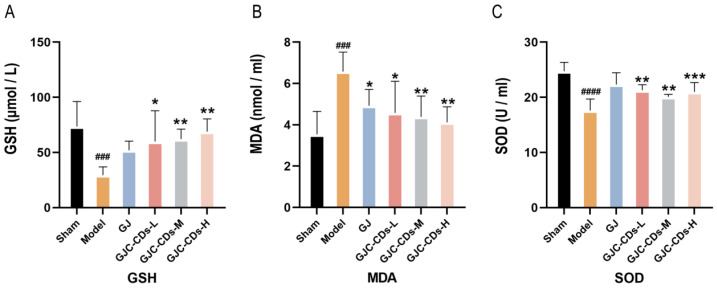
Effect of GJC-CDs on oxidative stress levels in MCAO mice. (**A**) GSH levels in serum (*n* = 6). (**B**) MDA levels in serum (*n* = 6). (**C**) SOD levels in serum (*n* = 6). Data were represented as means ± SD. ^###^ *p* < 0.001, ^####^ *p* < 0.0001 compared with the Sham group, * *p* < 0.05, ** *p* < 0.01, and *** *p* < 0.001 compared with the Model group.

Mitochondria have been demonstrated to be critical for maintaining energy homeostasis. The purpose of this study was to investigate whether the cerebroprotective effects of GJC-CDs are associated with an increase in the body’s antioxidant levels. Therefore, the content of oxidative stress levels was assessed in various groups of mice. As shown in Figure 7A–C, compared with the sham-operated group, the levels of GSH and SOD were significantly lower (*p* < 0.0001), while MDA was significantly higher in the model group (*p* < 0.0001), indicating that the mice were in a state of excessive oxidative stress after ischemic stroke. However, pre-administration of GJC-CDs (1.5, 3 and 6 mg/kg, respectively) significantly reduced MDA levels and resulted in an elevation in GSH and SOD levels in cerebral ischemic mice, with the most pronounced efficacy at the higher dose. Furthermore, the trend in the data showed that the GJ group improved the level of oxidative stress in mice, but GSH and SOD were not statistically different from the model group (*p* > 0.05).

### 2.7. LPS-Induced Inflammatory Response in BV2 Microglia Cells

**Figure 8:**
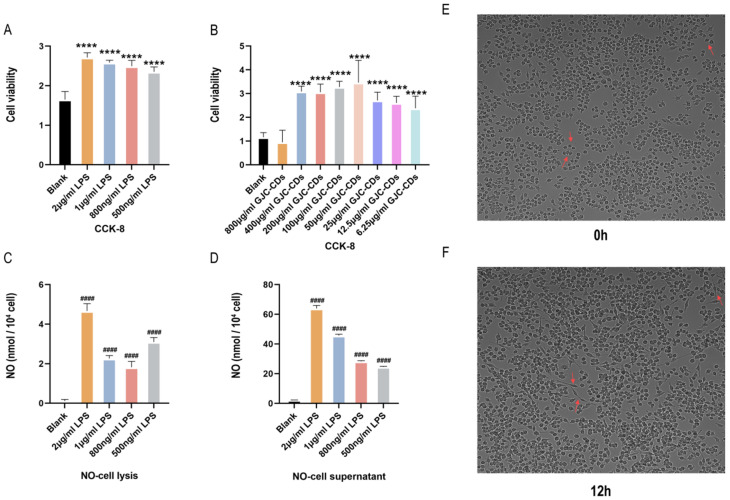
Effect of different concentrations of LPS in BV2 microglia cells. (**A**,**B**) CCK-8 (*n* = 6). (**C**) NO–cell lysis assay (*n* = 6). (**D**) NO–cell supernatant assay (*n* = 6). (**E**,**F**) Morphology of BV2 cells stimulated at different times at 2 μg/mL LPS (200×, arrows show morphological changes in BV2 microglia cells in response to LPS stimulation). Data were represented as means ± SD. ^####^ *p* < 0.0001 compared with the Blank group, **** *p* < 0.0001 compared with the Blank group.

As microglia serve as resident macrophages in the brain and play a pivotal role in initiating defense responses against infections or neuroinflammation, BV2 microglia cells were utilized to investigate the anti-neuroinflammatory effects of GJC-CDs. CCK-8 assays revealed that all tested concentrations of GJC-CDs (except 800 μg/mL) did not affect cell viability (Figure 8A,B). Although there was no statistical difference between the 800 μg/mL GJC-CDs group and the blank group, the trend of the data showed a significant decrease in severe cell viability at this drug concentration, probably because of the relatively small cytotoxicity of GJC-CDs present at this concentration. Therefore, we did not choose this concentration for subsequent cell experiments. To determine the optimal LPS concentration for modeling inflammation, nitric oxide (NO) levels were measured, as NO is a key inflammatory mediator produced by activated microglia via inducible nitric oxide synthase (iNOS). Given the instability of NO in culture systems, both cell supernatants and lysates were analyzed. Results (Figure 8C,D) demonstrated that 2 μg/mL LPS significantly increased NO levels. High-content imaging (Figure 8E,F) further confirmed that BV2 cells exhibited activated amoeboid morphology after 12 h of stimulation with 2 μg/mL LPS. This concentration was selected for subsequent experiments.

### 2.8. Effect of GJC-CDs on LPS-Induced Inflammatory Mediators and Oxidative Stress in BV2 Microglia Cells

We preliminarily screened the dosing concentrations for the subsequent experiments by examining the levels of NO. We first chose to administer concentrations of 400, 200, 100, and 50 µg/mL GJC-CDs and found that the best results were achieved with the administration of 50 µg/mL. Then, we continued to dilute the drug concentration downward and chose the concentrations of 100, 50, 25, and 12.5 µg/mL GJC-CDs and found that 50 µg/mL was still the most effective, the 25 and 12.5 µg/mL were better than 400 and 200 µg/mL. Therefore, we finally chose the concentrations of 100, 50, 25, and 12.5 µg/mL GJC-CDs for the subsequent experiments.

**Figure 9:**
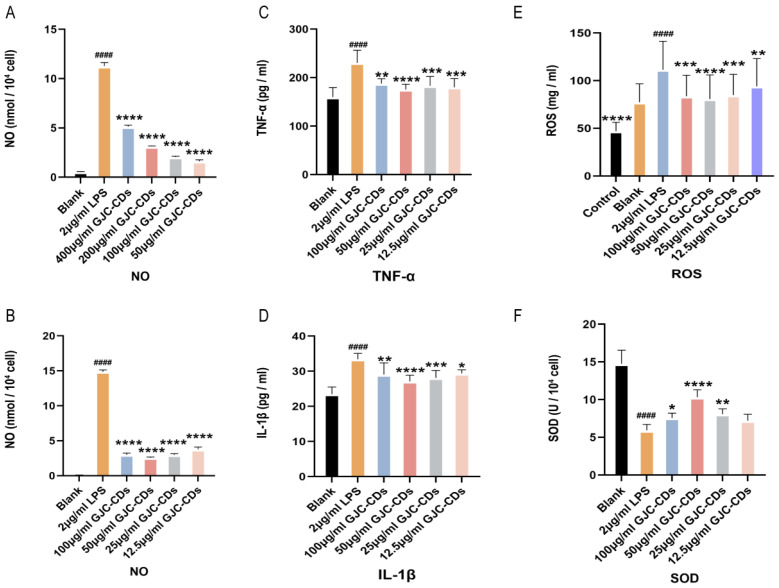
Effect of GJC-CDs on LPS-induced inflammatory mediators and oxidative stress in BV2 microglia cells. (**A**,**B**) NO levels in supernatant (*n* = 6). (**C**) TNF-α levels in supernatant (*n* = 6). (**D**) IL-1β levels in supernatant (*n* = 6). (**E**) ROS fluorescence intensity. (**F**) SOD levels in supernatant (*n* = 6). Data were represented as means ± SD. ^####^ *p* < 0.0001 compared with the Blank group; * *p* < 0.05, ** *p* < 0.01, *** *p* < 0.001, and **** *p* < 0.0001 compared with the 2 μg/mL LPS group.

We then determined the expression levels of inflammatory mediators (including IL-1β and TNF-α) in BV2 cells pretreated with different concentrations of GJC-CDs (12 h) and LPS (2 μg/mL, 12 h) by enzyme-linked immunosorbent assay. Results indicated that GJC-CD pretreatment significantly reduced pro-inflammatory cytokine levels in LPS-stimulated cells, with 50 μg/mL GJC-CD pretreatment showing the most potent inhibitory effects (Figure 9A–D). ROS levels were quantified using the DCFH-DA fluorescent probe (excitation/emission: 488/525 nm). LPS stimulation (2 μg/mL) markedly increased ROS fluorescence intensity, which was attenuated by GJC-CD pretreatment (Figure 9E), suggesting alleviation of LPS-induced oxidative stress. Additionally, the activity of superoxide dismutase (SOD), an antioxidant enzyme, was significantly reduced in LPS-treated cells but restored by GJC-CDs (Figure 9F). The results of the data from ROS and SOD assays also indicated that 50 μg/mL GJC-CDs pretreatment modulated oxidative stress best.

## 3. Discussion

The first report of carbon dots (CDs) synthesis was registered in 2004 as a new member of the carbon family and has attracted much attention from scientists over the last 20 years because of their advantageous properties, such as easy surface modification, extremely high water solubility, inexpensive and green synthetic methods, low toxicity, and excellent optical characteristics [23]. Due to these excellent properties, CDs have been extensively applied in different kind of scientific disciplines. For example, in the disease diagnosis, photocatalytic reactions, drug delivery, biological imaging, and photothermal and photodynamic as well as biological and chemical sensing therapies. Currently, relevant studies on CDs are still in their infancy, and their potential bioactivities have yet to be explored. In response to this current situation, it is worthwhile to develop a CD with novel bioactivities to advance its wide application in biomedicine.

The extraction of CDs from natural plants has proven to be a favored method amongst researchers, primarily due to the environmentally friendly, straightforward, and cost-effective nature of the process [24]. The utilization of plant-derived CDs, which were prepared by means of high-temperature pyrolysis, has been demonstrated to result in the manifestation of efficient biological activities. In comparison with the conventional methodologies that are commonly employed in this field, the present method has been shown to exhibit several distinct advantages, including a reduced reaction time, elevated temperature and an augmented yield [25]. Moreover, the resultant CDs particles have been observed to be distributed in a uniform manner, with smaller particle sizes, which has been identified as a key factor in their wide range of applications within the pharmaceutical field.

Medicinal plants, owing to their rich bioactive constituents, constitute an appropriate natural source for the synthesis of CDs with tailored physicochemical properties [26]. To the best of our knowledge, this study revealed for the first time a novel CDs with high bioactivity against ischemic stroke, which were roasted by the high-temperature green calcination method at 360 °C for 1 h to obtain GJC-CDs. *G. jasminoides* as the only plant source for the synthesis of GJC-CDs, is a traditional Chinese medicine with the advantages of being environmentally friendly, widely available, inexpensive, and abundant in biological constituents. CDs primarily consist of sp^2^-hybridized carbon with a surface abundant in functional groups such as oxygen, hydroxyl, and carboxyl groups [27]. These functional groups are likely to be one of the important reasons why GJC-CDs remain biologically active. We performed an optical analysis of the GJC-CD, which allowed the acquisition of their infrared, fluorescence, and ultraviolet spectrograms, as well as highly reactive functional groups such as carboxyl, hydroxyl, and amino groups. These results further elucidated the reason for their significant anti-ischemic stroke bioactivity.

A plethora of studies have evidenced the existence of three distinct categories of nanoparticle-mediated therapies for the treatment of ischemic stroke: neuroprotection, recanalization, and combination therapy [28,29,30]. The utilization of nanotechnology confers several advantages, including enhanced therapeutic effects, increased drug utilization, and reduced toxicity. These benefits are attributable to the regulated nanoscale, the ability to traverse the BBB, and the capacity to control the targeting of therapeutic drugs in specific environments [22]. In this study, it was found that pretreatment with GJC-CDs increased neurobehavioral scores, decreased the area of cerebral infarction, improved cerebral blood flow, and ameliorated pathological injury in the infarcted brain area, which indicated that GJC-CDs could attenuate cerebral ischemia–reperfusion injury. In exploring the protective mechanisms of GJC-CDs, we focused on their inhibition of the inflammatory response and excessive oxidative stress that occurs during ischemic stroke.

The nervous and immune systems work in tandem to maintain systemic homeostasis and respond to ischemic stroke in a coordinated series of responses. Transient cerebral ischemia and hypoxia, along with the subsequent neuronal cell death that ensues, activate resident immune cells or infiltrating peripheral immune cells [31]. This activation triggers local or even systemic inflammation, which profoundly affects the functional prognosis after stroke. The presence of inflammatory immune cells following cerebral ischemia has been demonstrated to exacerbate ischemic neuronal injury [32]. Consequently, the suppression of the inflammatory response following an ischemic stroke has emerged as a promising therapeutic strategy for the recovery of acute stroke. In this study, 24 h after central nervous system (CNS) ischemia, the concomitant inflammatory cascade response with elevated levels of tumor necrosis caused systemic peaks of TNF-α, IL-1β, and IL-6, and pre-administration of GJC-CDs reduced the levels of related inflammatory factors [33,34,35]. Furthermore, an increase in the anti-inflammatory factor IL-10 was observed in mice following administration. It can be concluded that GJC-CDs have the capacity to reduce the inflammatory response induced by ischemia and hypoxia, which is beneficial to enhancing ischemic tolerance and disrupting the vicious cycle.

It is widely accepted that oxidative stress is a significant causative factor for brain damage in cases of ischemic stroke. When a stroke occurs, the body produces ROS, leading to excessive oxidative stress [36]. Subsequently, cerebral ischemia causes peroxidation of proteins, lipids, and nucleic acids, leading to DNA damage and mitochondrial dysfunction and ultimately neuronal death. SOD and MDA are significant indicators of oxidative stress, representing antioxidant enzyme activity and oxygen radical lipid peroxidation, respectively [37]. Furthermore, GSH protects cell membranes from oxidative damage by reducing hydrogen peroxide and lipids [38]. The results of the study demonstrated that the GJC-CDs intervention significantly increased the total antioxidant capacity, up-regulated the levels of SOD and GSH, and down-regulated the levels of MDA. The inhibitory effect of GJC-CDs on oxidative stress during stroke may be due to its ability to scavenge free radicals, suggesting that GJC-CDs are able to reduce ROS, inhibit free radical lipid peroxidation, and attenuate ischemic brain damage. The results of the present study suggest that GJC-CDs from natural plants have the potential to be a promising strategy for anti-ischemic stroke therapy.

The biosafety of CDs has been proven to be a significant impediment to clinical application and promotion. Its metabolic disorders and side effects may affect normal physiological functions. Therefore, relevant biosafety experiments should be carried out for evaluation [39].

The results of cellular experiments showed that GJC-CDs pretreatment significantly increased BV2 cell viability and exhibited low cytotoxicity only at higher concentrations (800 µg/mL). Furthermore, GJC-CDs also increased the anti-inflammatory and antioxidant capacity of the BV2 cells. This is mainly due to the presence of abundant surface-carrying functional groups, which provide fundamental biosafety information for further investigation of the biological activities of GJC-CDs. The above results indicate that GJC-CDs has potential application prospects in the biomedical field.

Current therapeutic strategies for stroke generally comprise dilatation, thrombolysis, and neuronal protection, but are frequently accompanied by a range of adverse side effects. The effects of GJC-CDs against cerebral ischemia–reperfusion injury have been shown, and their mechanism of action may be related to increasing the anti-inflammatory and antioxidant levels of the body. Thus, this new type of CD may be suitable for the treatment of ischemic stroke patients.

## 4. Materials and Methods

### 4.1. Chemicals

*G. jasminoides* (dried and matured fruits of the herb) was purchased from Beijing Qiancao Herbal Pieces Co., Ltd. (Beijing, China) and had passed quality verification by the National Medical Products Administration (Beijing, China). Cell counting kit (CCK-8) and other analytical grade chemical reagents were obtained from Beijing BioDee Biotechnology Co., Ltd. (Beijing, China). Dialysis bags were provided by Beijing Ruida Henghui Technology Development Co., Ltd. (Beijing, China). Enzyme-linked immunosorbent assay (ELISA) kits were purchased from Jiangsu Kete Biotechnology Co., Ltd. (Beijing, China). SOD, MDA, and GSH Biochemical kits for animal assays were purchased from Nanjing Jiancheng Bioengineering Institute (Nanjing, China). SOD and ROS Biochemical kits for cell assays were purchased from Addison Biotechnology Co., Ltd. (Yancheng, China). All the experiments were conducted using deionized water.

### 4.2. Animals

All the experimental procedures were performed in agreement with the Regulations for the Administration of Affairs Concerning Experimental Animals approved by the State Council of People’s Republic of China. The experimental procedures were approved by the Committee of Ethics of Animal Experimentation of Beijing University of Chinese Medicine (BUCM) and by the Health Guide for Care and Use of Laboratory Animals. Male C57BL/6J mice (20~25 g) purchased from Beijing Sibeifu Experimental Animal Technology Co., Ltd. (Beijing, China) were housed in a laboratory environment with constant temperature (24.0 ± 1.0 °C) and relative humidity (45.0–55.0%) on a 12 h light/dark cycles and allowed to feed ad libitum. The project identification code for this batch of mice is BUCM-2024111506-4290. The date of approval is 15 November 2024.

### 4.3. Preparation of GJC-CDs

Briefly, a green and safe one-step pyrolysis method was used to prepare GJC-CDs from *G. jasminoides*. To begin with, 300 g of *G. jasminoides* is divided into different crucibles and sealed with fixed tops with tinfoil to make a tight fit seal, at this time, simmering calcination in a 70 °C muffle furnace (TL0612, Beijing Zhong Ke Anbo Innovation Co., Ltd., Beijing, China) for 30 min; in the second stage, the temperature of the muffle furnace is set to 360 °C, simmering calcination is performed for 1 h, then the *G. jasminoides* charcoal (GJC) is finally obtained. After the temperature has cooled to room temperature, GJC was crushed with a micro pulveriser. The fine powders of GJC (100 g) were mixed and dispersed in 1000 mL DW, followed by boiling at 100 °C three times for 1 h in a thermostatic water bath. The resulting solution was subjected to crude filtration using a Brinell funnel, then the filtrate was further subjected to fine filtration using a 0.22 μm cellulose acetate membrane; finally, the resulting solution was purified by dialysis using a 1.0 kDa dialysis membrane for 7 days until the color of the solution in the dialysis bag was not changing. Multiple centrifugations were performed to remove the residue to obtain GJC-CD dialysate, which was freeze-dried to powder and stored at 4 °C in the refrigerator.

### 4.4. Characterization of GJC-CDs

The particle size distribution, microstructure, and atomic lattice fringes of GJC-CDs was observed by a high-resolution transmission electron microscope (HRTEM, 200 kV, JEOL, Tokyo, Japan) and transmission electron microscope (TEM, 100 kV, Talos F200X-G2, Hillsborough, NC, USA).

The surface elemental composition of GJC-CDs was observed by using the X-ray photoelectron spectroscopy (XPS, ESCALAB 250 Xi; Termo Fisher Scientific, Waltham, MA, USA) with Al Kα excitation (1486.6 eV) as the X-ray source and a Fourier transform-infrared (FT-IR) spectrometer (Termo Fisher, Waltham, MA, USA) at the range of 400–4000 cm^−1^. XRD (D8-Advanced X-ray diffractometer, Bruker AXS, Karlsruhe, Germany) was performed with Cu K-alpha radiation. In addition, using fluorescence (F-4500, Tokyo, Japan) and UV-Vis (CECIL, Cambridge, UK) spectroscopy, respectively, the fluorescence and ultraviolet-visible spectrum characteristics were observed and recorded.

### 4.5. Animal Experimental Protocol

A total of 150 C57BL/6J mice were randomly divided into 6 groups (*n* = 25, in each group), and the protocol was administered as follows:

Sham group (Sham): the animals were pre-administered with saline solution for 7 days and underwent the same surgery without MCAO on the 8th day.

Model group (Model): the animals were pre-administered with saline solution for 7 days and underwent MCAO surgery on the 8th day.

*G. jasminoides* pretreatment group (GJ): the animals were pre-administered with 10 g/kg/d of *G. jasminoides* aqueous extract for 7 days and underwent MCAO surgery on the 8th day.

Low-dose GJC-CDs pretreatment group (L): the animals were pre-administered with 1.5 mg/kg/d of GJC-CDs for 7 days and underwent MCAO surgery on the 8th day.

Medium-dose GJC-CDs pretreatment group (M): the animals were pre-administered with 3 mg/kg/d of GJC-CDs for 7 days and underwent MCAO surgery on the 8th day.

High-dose GJC-CDs pretreatment group (H): the animals were pre-administered with 6 mg/kg/d of GJC-CDs for 7 days and underwent MCAO surgery on the 8th day. The idea behind the design of the GJ group was to investigate how the bioactivity of GJC-CDs extracted from *G. jasminoides* compared to that of the *G. jasminoides* decoction and to investigate whether GJC-CDs have higher bioactivity than the traditional *G. jasminoides* decoction.

Mice in all groups were given saline solution or drugs by gavage.

Mice in *G. jasminoides* and GJC-CDs pretreatment groups underwent MCAO for 60 min followed by reperfusion. In addition, mice in the sham group underwent the same surgical procedures, except that the silicone-coated nylon suture was not inserted into the middle cerebral artery. Twenty-four h after reperfusion of the animals in each group, the degree of neurological impairment was assessed and quantified in all mice according to the Zea-Longa scale, which was divided into five grades with a score of 0–4. When the score was 0 or 4, or when mice were found to be dead before the scoring, the mice were excluded and were not used in the subsequent experiments.

### 4.6. Middle Cerebral Artery Occlusion

The MCAO model was induced by intraluminal suture occlusion of the middle cerebral artery [40]. Initially, the animals were anesthetized with rumpun barbituin natrium (1%, 10 mL/kg). The external carotid artery (ECA), internal carotid artery (ICA), and common carotid artery (CCA) were isolated in mice. All of the above vessels were bluntly separated after exposure through a midline neck incision. Subsequently, the external carotid artery (ECA) is ligated, and the silicon-coated nylon suture is inserted into the internal carotid artery (ICA) about 8–10 mm under the guidance of a visualizer until it reaches the proximal part of the anterior cerebral artery (ACA), thus blocking the origin of the middle cerebral artery (MCA). After 1 h of ischemia, the silicon-coated nylon suture was removed, and blood was reperfused for 24 h.

### 4.7. Neurological Defcit Score

The mice in each group were scored using Zea-Longa grading criteria after 1 h of ischemia and 24 h of reperfusion [41]. The neurobehavioral scoring criteria were as follows: 0, mice behaved normally without neurological impairment; 1, the right forelimb was flexed and inwardly retracted when the tail of the mouse was lifted; 2, the mice rotated to the right side when they were horizontally crawling; 3, the mice tilted to the right side when they stood up or crawled; 4, the mice suffered from impaired consciousness accompanied by loss of autonomic activity behaviors.

### 4.8. Assessment of Cerebral Blood Flow

Laser scatter hemodynamic imaging was employed to detect alterations in cerebral blood flow in each group of mice. Firstly, the entire brain within the cranium of the mice was exposed, and the blood flow within the bilateral brains of the mice was detected using the laser scatter blood flow video detection system. The recorded cerebral blood flow changes were observed for a period of 30 s. Subsequently, the laser scatter blood flow contrast maps of the brains of mice in each group were assembled for the purpose of analyzing the alterations in cerebral blood flow within the cerebral cortex and the imaging characteristics of the mice throughout the monitoring period. Ultimately, the ratio of cerebral blood flow on the infarcted side to cerebral blood flow (CBF) on the healthy side was calculated based on the aforementioned measurements and subsequently subjected to statistical analysis.

### 4.9. Measurement of Infarct Volume

The volume of cerebral infarction in mice after MCAO was detected using 2,3,5-triphenyltetrazolium chloride (TTC) staining as follows: firstly, the mouse brain tissue was quickly dissected, and then the frozen brain tissue was cut into brain slices of approximately 2 µm thickness. The brain slices from each group of mice were then placed in a box containing 2% TTC solution for staining, and after the TTC staining was completed, the brain slices were transferred to 4% paraformaldehyde for fixation for 24 h and finally photographed for analysis. TTC stained grayish white are infarcted areas (IS) and red are non-infarcted areas (NIS).

Area of cerebral infarction (%) = area of cerebral infarction/area of whole brain × 100%

### 4.10. Histopathological Examination of the Brain

At 24 h after reperfusion, mice were perfused transcardially, first with 0.9% saline and then with ice-cold 4% paraformaldehyde. Fresh brain tissues were fixed in paraformaldehyde for 24 h prior to tissue sectioning. The brain tissue sections of each group of mice were then dehydrated using an ethanol gradient, embedded in paraffin, stained with both hematoxylin eosin (HE) staining reagent and Nissl staining reagent (toluidine blue). Following a second dehydration step, the tissues were transparentized. Finally, the pathological morphology of the brain tissues of the mice was observed and photographed, and the results were analyzed under a light microscope.

### 4.11. Serum Inflammatory Factor Analysis

The mice were anesthetized, and blood was collected and centrifuged at 3000 r·min^−1^ for 20 min at 4 °C to obtain serum. The ELISA kits purchased from Jiangsu Kete Biotechnology Co., Ltd. Were used to measure IL-6 (3–120 pg/mL), IL-10 (30–1000 pg/mL), IL-1β (2.5–80 ng/mL), and TNF-α (25–800 ng/mL) levels in accordance with the instructions provided by the manufacturer. The results of these indicators were obtained by measuring the absorbance of each well at a wavelength of 450 nm.

### 4.12. Assessment of Oxidative Stress in Serum

The mice were subjected to anesthesia, after which blood samples were taken and centrifuged at 3000 r.min^−1^ for 20 min at a temperature of 4 °C to separate the serum. Following this, the serum concentrations of SOD (2–50 U/mL), MDA (0.5–113 nmol/mL), and GSH (0–100 μmol/L) were determined using the microplate assay, as per the manufacturer’s guidelines. The detection results of SOD were obtained by measuring the absorbance at a wavelength of 450 nm. The detection results of MDA were obtained by measuring the absorbance at a wavelength of 532 nm. The detection results of GSH were obtained by measuring the absorbance at a wavelength of 405 nm.

### 4.13. Cell Culture and Treatment

The BV2 cell line was obtained from the Cell Resource Center of the Institute of Basic Medical Sciences, Chinese Academy of Medical Sciences. BV2 cells were routinely cultured in a standard incubator (37 °C, 5% CO_2_, unrestricted O_2_) using complete medium (DMEM supplemented with 10% FBS). They were pre-inoculated into 6-well plates prior to experimentation. After 24 h of acclimatization, BV2 cells were pretreated with varying concentrations of GJC-CDs for 12 h to assess their anti-inflammatory effects. Subsequently, cells were stimulated with lipopolysaccharide (LPS) for 12 h, followed by corresponding treatments based on specific experimental indicators.

### 4.14. Cell Viability Assay

The safety of carbon nanodots (GJC-CDs) and LPS was evaluated using the standard CCK-8 assay. Briefly, BV2 cells were harvested and seeded into 96-well plates at a density of 1 × 10^5^ cells per well. After 24 h of acclimatization, cells were treated with GJC-CDs (6.25, 12.5, 25, 50, 100, 200, 400, and 800 μg/mL diluted in complete medium) or LPS (500, 800, 1000, 2000, and 5000 ng/mL diluted in complete medium). Cells treated with complete medium alone served as vehicle controls. After 24 h of incubation, 10 μL of CCK-8 solution was added to each well. The plates were further incubated at 37 °C for 3 h, and absorbance was measured at 450 nm using a microplate reader.

### 4.15. Detection of Inflammatory Factors in Cell Supernatants

The BV2 microglia cells were centrifuged at 3000 r.min^−1^ for 10 min at 4 °C to obtain cell supernatant. The ELISA kits purchased from Meibiao Biology Biotechnology Co., Ltd. (Yancheng, China) were used to measure IL-1β (3.75–120 pg/mL) and TNF-α (1–640 pg/mL) levels in accordance with the instructions provided by the manufacturer. The results of these indicators were obtained by measuring the absorbance of each well at a wavelength of 450 nm.

### 4.16. ROS Detection

Intracellular reactive oxygen species (ROS) levels were measured using a ROS Assay Kit (AIDISHENG, Yancheng, China). Cells seeded in 6-well plates were pretreated with GJC-CDs (12.5, 25, 50 and 100 μg/mL) for 12 h, followed by LPS (2 μg/mL) stimulation for another 12 h. After treatment, cells were washed twice with serum-free medium and incubated with 2′,7′-dichlorodihydrofluorescein diacetate (DCFH-DA) (final concentration: 10 μM) in the dark for 20 min. Cells were then stained with Hoechst 33342 (1 μg/mL) for 10 min, washed twice with serum-free medium, and ROS levels were quantified using a microplate reader at excitation/emission wavelengths of 488 nm/525 nm, respectively. The kit provides a positive control reagent (Rosup), at a concentration of 50 mg/mL.

### 4.17. Statistical Analysis

All data in this study were statistically analyzed by IBM SPSS Statistics (version 26). The mean ± standard deviation (SD) indicated that the data exhibited a normal distribution. Subsequently, differences between groups were performed by one-way ANOVA followed by the least significant difference test or Dunnett’s T3 method. Additionally, the comparison of non-normal distributed data was evaluated by the Kruskal–Wallis H test. In this study, * *p* < 0.05, ** *p* < 0.01, and *** *p* < 0.001 were considered statistically significant.

## 5. Conclusions

Novel green GJC-CDs were synthesized by the one-step calcination method without adding any reagent using natural plant *G. jasminoides* as a raw material, and their biosafety was explored by cell viability experiments. The purified GJC-CDs, with regular size and abundant chemical groups, showed significant protective effects in an animal model of ischemia–reperfusion injury, which was preliminarily demonstrated to be associated with anti-inflammatory and antioxidant properties. This study offers promising potential for GJC-CDs as a viable alternative to current clinical prevention strategies for ischemic stroke.

## Data Availability

Data are contained within the article.

## Abbreviations

The following abbreviations are used in this manuscript:

IS	ischemic stroke
CDs	carbon dots
GJ	*Gardenia jasminoides*
GJC-CDs	*Gardenia jasminoides* carbonisata
MCAO	middle cerebral artery occlusion
DAMP	damage-associated molecular patterns
ROS	reactive oxygen species
MPTP	mitochondrial permeability transition pore
rtPA	recombinant tissue plasminogen activator
BFGF	basic fibroblast growth factor
TNF-α	tumor necrosis factor-α
IL-1β	interleukin-1β
IL-6	interleukin-6
IL-10	interleukin-10
GSH	glutathione
SOD	superoxide dismutase
MDA	malondialdehyde
CCK-8	cell counting kit-8
CBF	cerebral blood flow
TTC	2,3,5-triphenyltetrazolium chloride
HE	hematoxylin eosin
